# RETRACTION: The Effect of Nursing Intervention Based on the Staged Behaviour Change on Recovery, Quality of Life, and Self‐Efficacy of Diabetic Patients With Scalds

**DOI:** 10.1111/iwj.70653

**Published:** 2025-04-23

## Abstract

**RETRACTION:** LiC. , ChenZ. , GaoB. , YangM. , RenL. , LiJ. , ZhangY. , and YangM. , “The Effect of Nursing Intervention Based on the Staged Behaviour Change on Recovery, Quality of Life, and Self‐Efficacy of Diabetic Patients With Scalds,” International Wound Journal 19, no. 1 (2022): 202–210, 10.1111/iwj.13622.34080304 PMC8684861

The above article, published online on 2 June 2021, in Wiley Online Library (http://onlinelibrary.wiley.com/), has been retracted by agreement between the journal Editor in Chief, Professor Keith Harding; and John Wiley & Sons Ltd. Following an investigation by the publisher, all parties have concluded that this article was accepted solely on the basis of a compromised peer review process. The editors have therefore decided to retract the article. The authors did not respond to our notice regarding the retraction.

**RETRACTION:** C. Li , Z. Chen , B. Gao , M. Yang , L. Ren , J. Li , Y. Zhang , and M. Yang , “The Effect of Nursing Intervention Based on the Staged Behaviour Change on Recovery, Quality of Life, and Self‐Efficacy of Diabetic Patients With Scalds,” International Wound Journal 19, no. 1 (2022): 202–210, 10.1111/iwj.13622.34080304 PMC8684861

The above article, published online on 2 June 2021, in Wiley Online Library (http://onlinelibrary.wiley.com/), has been retracted by agreement between the journal Editor in Chief, Professor Keith Harding; and John Wiley & Sons Ltd. Following an investigation by the publisher, all parties have concluded that this article was accepted solely on the basis of a compromised peer review process. The editors have therefore decided to retract the article. The authors did not respond to our notice regarding the retraction.

